# Supplementary figures and images for: Direct male development in chromosomally ZZ zebrafish
Source: Front Cell Dev Biol. 2024 Mar 11;12:1362228. doi: 10.3389/fcell.2024.1362228 (PMC10961373; doi:10.3389/fcell.2024.1362228)

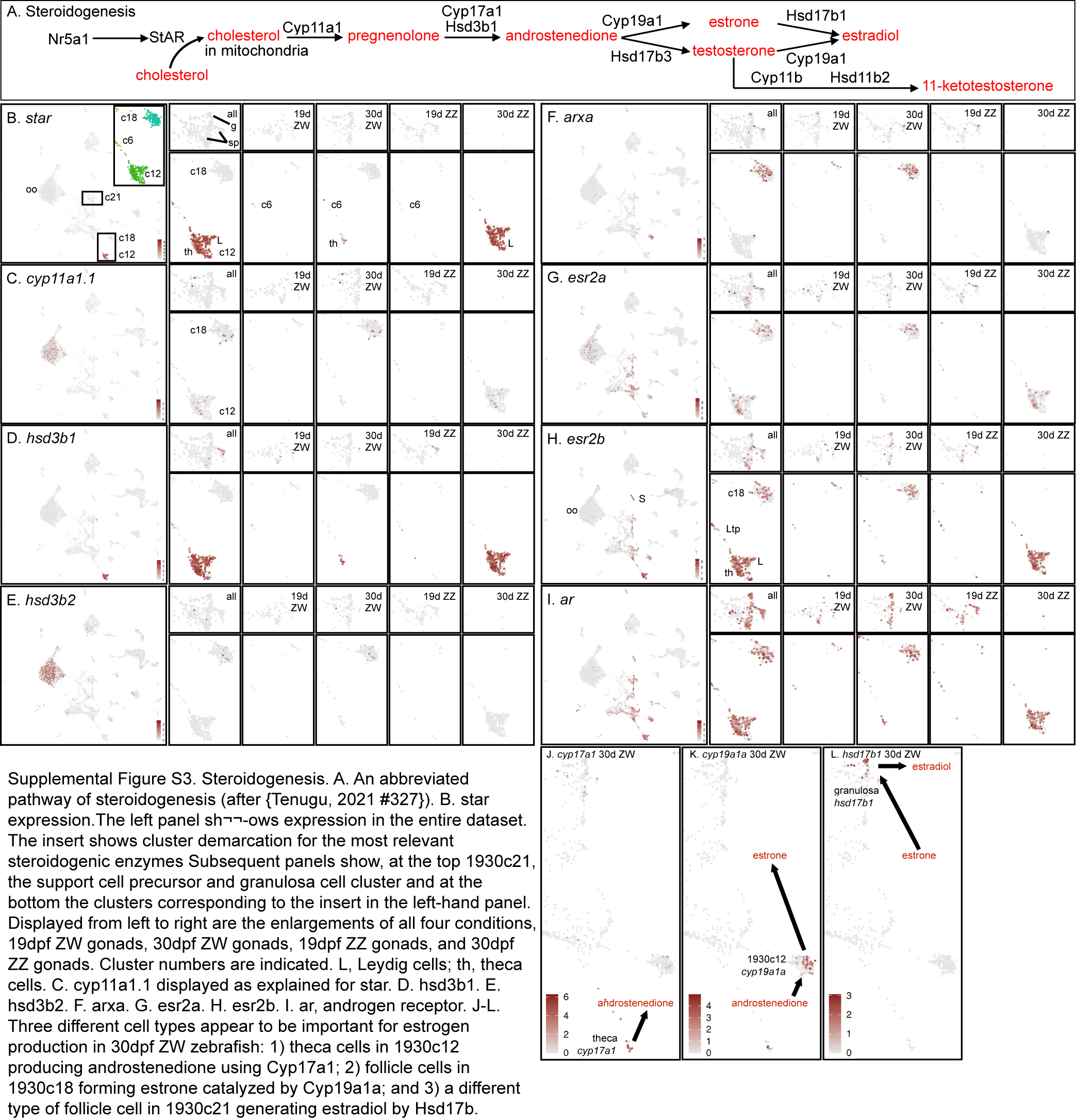

Supplement: Supplementary file 3 [file Image3.TIF]

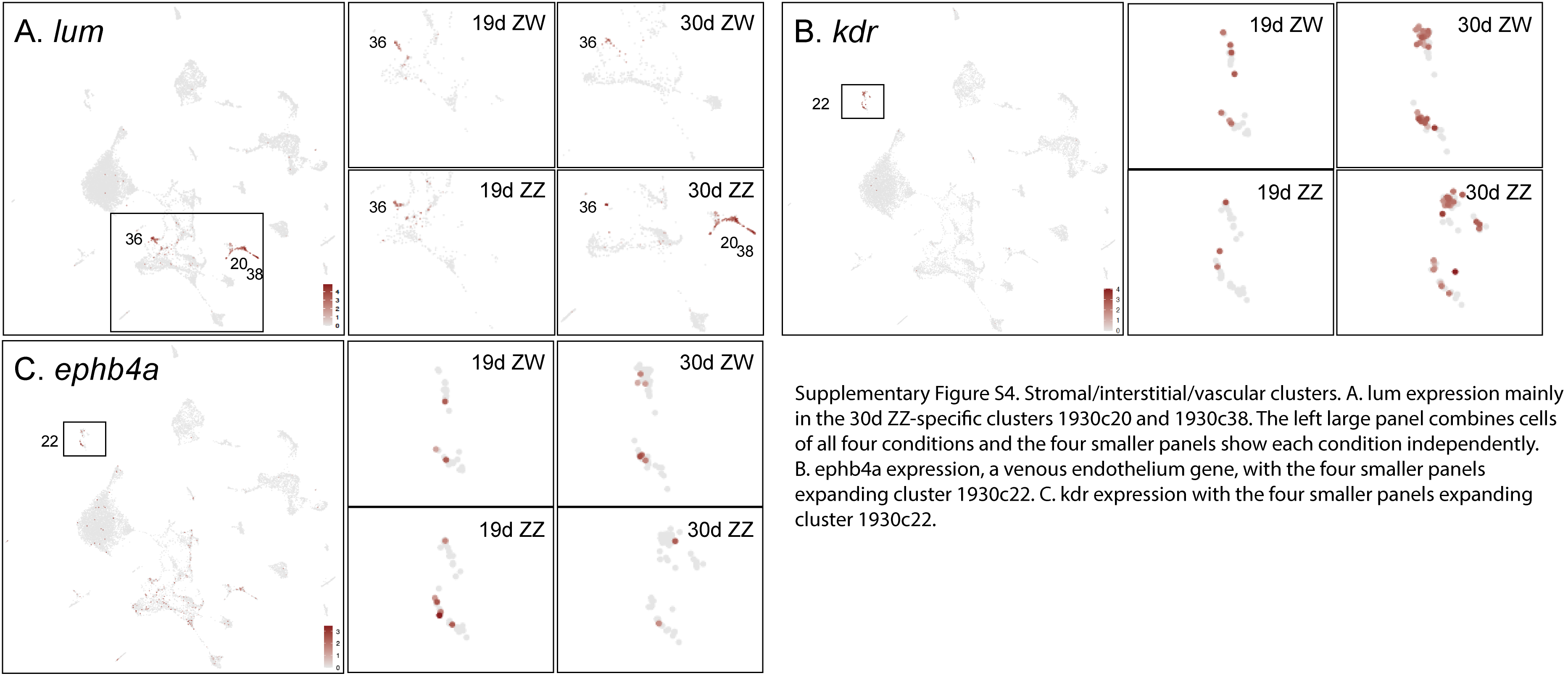

Supplement: Supplementary file 4 [file Image4.TIF]

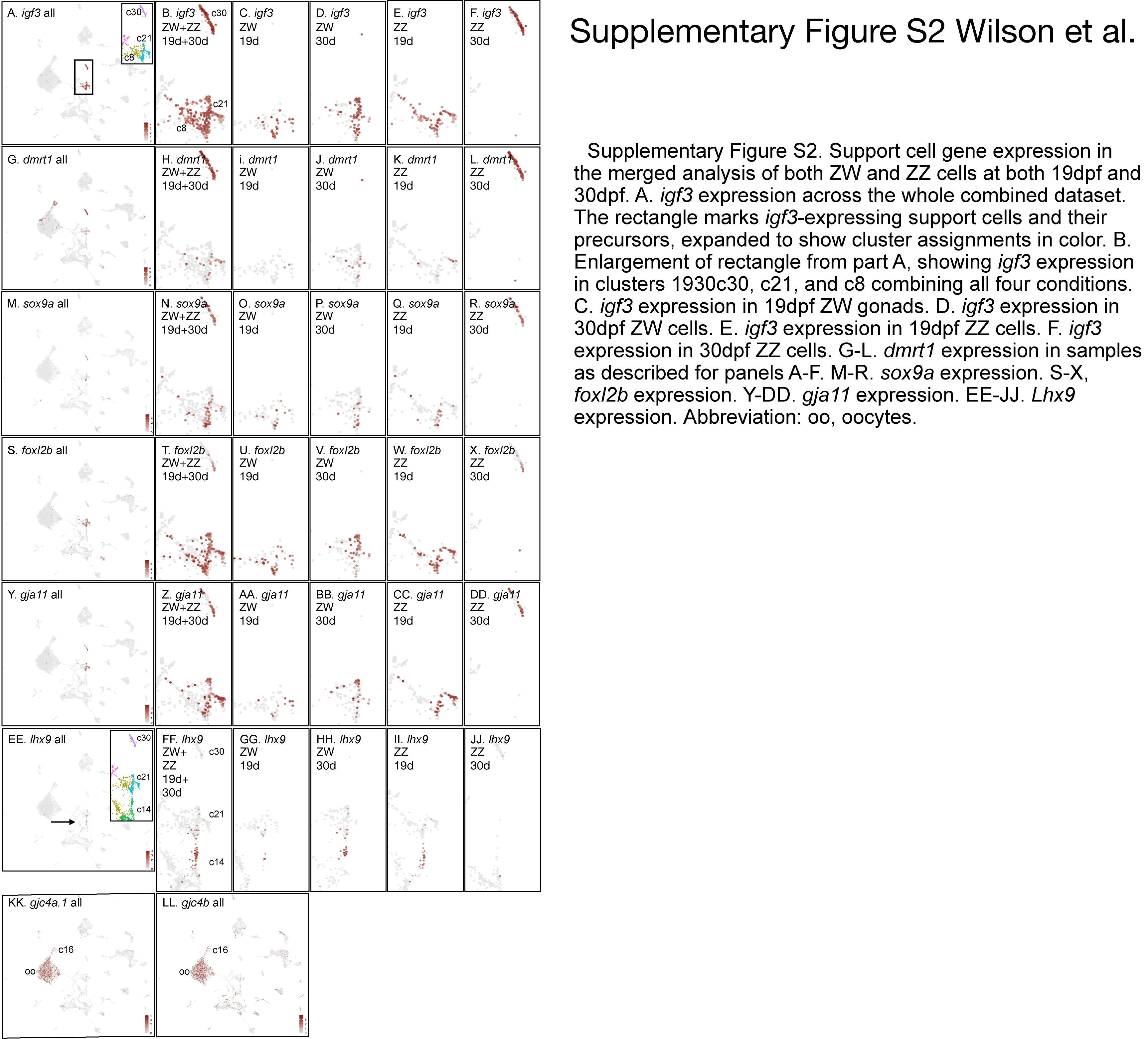

Supplement: Supplementary file 5 [file Image2.TIF]

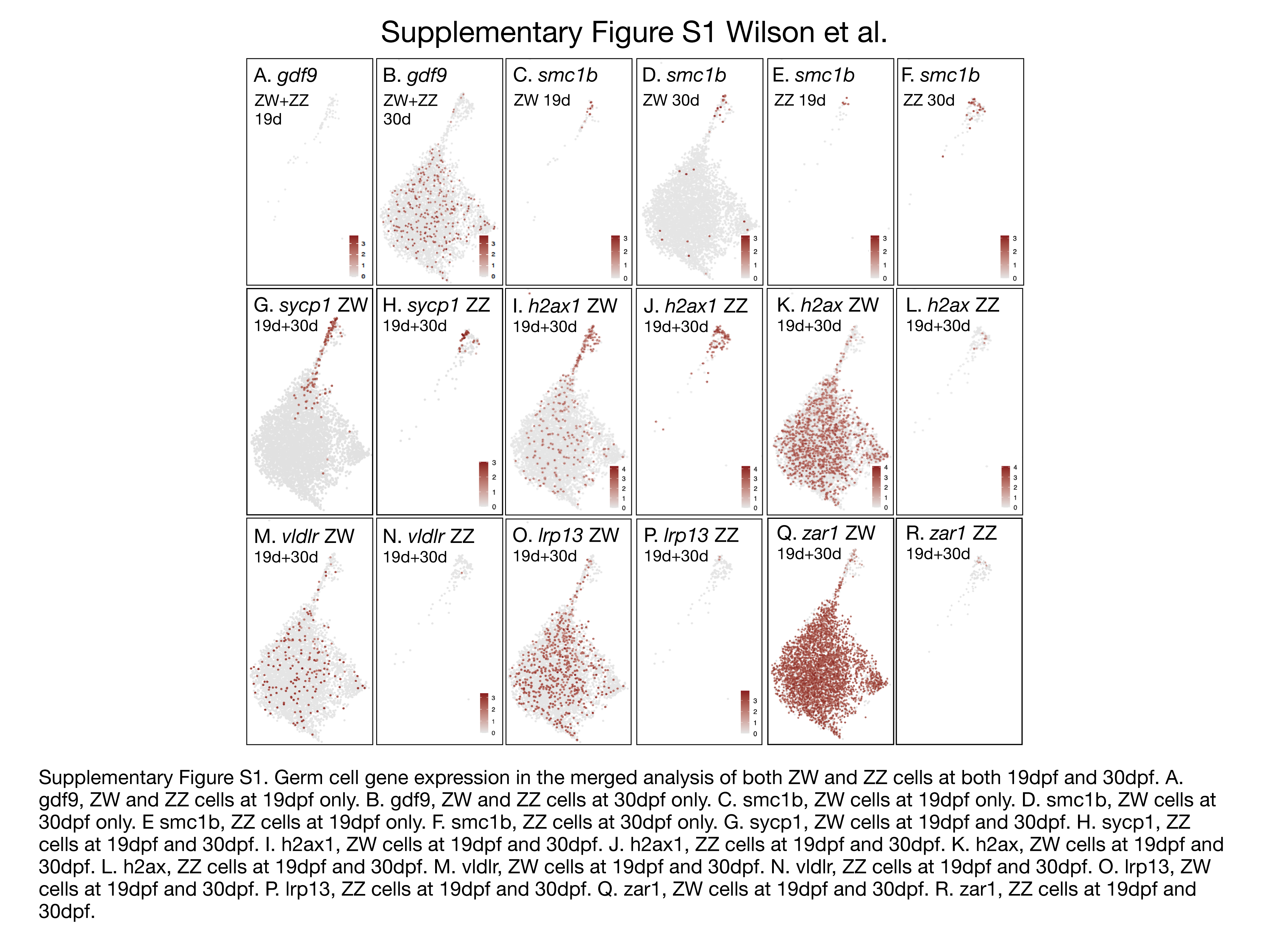

Supplement: Supplementary file 6 [file Image1.TIF]
